# Reliability and sensitivity analysis of double inverted-T nano-cavity label-free Si:HfO_2_ ferroelectric junctionless TFET biosensors

**DOI:** 10.1039/d2ra03118c

**Published:** 2022-09-26

**Authors:** Sangeeta Singh, Shradhya Singh, Mustafa K. A. Mohammed, Kamal Kishor Jha, Sajad A. Loan

**Affiliations:** Microelectronics & VLSI Lab, National Institute of Technology Patna-800005 India sangeeta.singh@nitp.ac.in; Department of Medical Physics, Al-Mustaqbal University College 51001 Hillah Babylon Iraq; Indian Institute of Information Technology Vadodara India; Jamia Millia Islamia New Delhi India

## Abstract

In this work, we propose and simulate an ultrasensitive, label-free, and charge/dielectric modulated Si:HfO_2_ ferroelectric junctionless tunnel field effect transistor (FE-JL-TFET) based biosensor. The proposed sensing device employs a dual inverted-T cavity and uses ferroelectric gate stacking of Si-doped HfO_2_, a key enabler of negative capacitance (NC) behavior. The two cavities are carved in gate-source underlap regions by a sacrificial etching technique to sense biomolecules such as streptavidin (2.1), bacteriophage T7 (6.3) and gelatin (12). Two dimensional (2D) calibrated simulations have been performed and the impact of various device parameters, including cavity length and height, on various performance measuring parameters has been studied. It has been observed that the biosensor exhibits better sensitivities for both neutral and charged biomolecules. The maximum values of the *I*_ON_/*I*_OFF_ sensitivity for the neutral, positively charged and negatively charged biomolecules are as high as 3.77 × 10^9^, 5.85 × 10^9^, and 1.72 × 10^10^, respectively. It has been observed that optimizing the cavity length and height can significantly improve the sensing capability of the proposed device. The comparative analysis of the proposed biosensor and other state of the art biosensors shows a significant improvement in the sensitivity (10^1^ to 10^6^ times) in the proposed biosensor. The detrimental effect of interface trapped charges on the biosensor performance is also analyzed in detail.

## Abstract 2

We propose and simulate an ultrasensitive, label-free, and charge/dielectric modulated Si:HfO_2_ ferroelectric junctionless tunnel field effect transistor (FE-JL-TFET) based biosensor.

## Graphical abstract

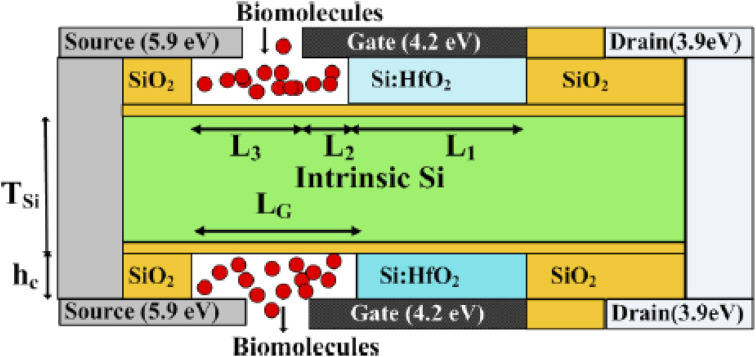

## 1 Introduction

The development of nanoscale ultrasensitive biosensors has revolutionized the medical science field by easy and early detection of various diseases like the current COVID-19.^1^ The International Union of Pure and Applied Chemistry (IUPAC) defines a biosensor as a device that detects chemical compounds *via* electrical, thermal, or optical signals released by isolated enzymes, tissues or cells.^2^ The various sensing techniques include test strip based electrochemical technologies, optical detection and the latest nanoelectronics based sensing technologies.^3–5^ The nanoelectronics sensing technologies employ field effect transistor (FET) based sensors with tremendous potential due to their rapid response time, scalability, a simpler recovery mechanism, and high sensitivity even at smaller channel lengths. In a FET based sensor, the sensing can be done by a cavity in the dielectric material or by employing an appropriate receptor over the gate dielectric material. The presence of biomolecules in the cavity or on the receptor over the gate dielectric material changes the ON current (*I*_ON_), threshold voltage shift and *I*_ON_/*I*_OFF_ ratio of the sensing device and hence can be easily detected. The first biosensor, an ion sensitive FET (ISFET) was developed by Befgveld in 1970. It shows better sensitivity for charged biomolecules, however, its response for neutral biomolecule detection is poor.^6,7^ Dielectric modulation FETs (DM-FETs) are satisfactory for detecting both neutral and charged biomolecules; however, their functionality is limited due to scaling, other short channel effects (SCEs) and larger detection time.^8^ Tunnel field effect transistors (TFETs) have resolved the scaling issues of FETs due to their steep subthreshold swing (SS) (below 60 mV per decade), lower leakage current and higher immunity towards SCEs, and low energy consumption devices.^9^ Various dielectrically modulated (DM) TFET sensing devices have been developed and extensively utilized for the label free (by definition, label-free biosensing systems don’t employ labels to make measurements easier. Instead, they use the analytes’ inherent physical characteristics, such as their size, charge, electrical impedance, dielectric permittivity, and refractive index, to identify them in a sample) detection of biomolecules owing to their better sensitivity and energy efficiencies.^10–16^ However, some of the prominent issues with TFET devices are low ON current, ambipolarity, the requirement for sharp junctions, RDFs, higher thermal budget *etc.* Abrupt junctions are crucial for band-to-band tunneling and hence address the ON current issue in TFETs. The concept of charge plasma/electrostatic doping based on work function engineering has been employed to address the RDF (a well-known phenomenon caused by the unpredictability of charge position and quantity, such as discrete placement of dopant atoms that follow a Poisson distribution in the channel region, and the overall number of channel dopants reduces as the device size decreases, leading to a greater variety in dopant quantities and a considerable influence on threshold voltage) and sharp junction issues in TFET based sensors^17–19^ as junctionless transistors have better scalability, simpler fabrication processes and reduced complexity. Furthermore, gate voltage and SS can be reduced in TFETs by using a ferroelectric (FE) material as a gate stack dielectric, which realizes an effective negative capacitance (NC) without sacrificing the ON current.^20–24^ Recently, many FE materials have been explored as gate stack dielectrics, like barium titanate (BaTiO_3_), lead zirconate titanate (PZT),^25,26^ and strontium bismuth tantalate (SBT), however, these materials have compatibility issues with Si and have severe scaling issues due to their large dielectric constants. Recent studies have shown that the Si doped HfO_2_ material has a small dielectric constant^27,28^ as compared to other ferroelectric materials, shows better scaling and supports the current technology nodes. Besides, the coercive field is as high as 1 MV cm^−1^ and shows good interface properties with silicon.^29,30^

The differential amplification of gate voltage at the silicon channel and gate oxide interface, and the stability of the hysteretic feature of the integrated FE gate stack, are the two most important design considerations for NCFETs. The negative capacitance effect of ferroelectric materials encourages low power design for biomolecule detection. In this work, we addressed the above mentioned issues of TFET biosensors by designing and simulating an ultra-sensitive, label-free, and dielectric modulated Si:HfO_2_ ferroelectric junctionless TFET (FE-JL-TFET). The proposed TFET biosensor employs dual inverted-T shaped nano-cavities carved beneath both gate-source electrodes to immobilize the biomolecules. When biomolecules with dielectric constant (*K*) such as streptavidin (2.1) (neutral),^27^ bacteriophage T7 (6.3) (positively charged)^28^ and gelatin (12) (negatively charged)^31^ enter into the cavities, the electrical characteristics of the device, such as *I*_ON_, *V*_th_ and SS will be accordingly altered and hence the corresponding biomolecule will be sensed. Further, the impact of device parameters including cavity length (*L*_c_) and cavity height (*h*_c_) on energy band, surface potential, drain current subthreshold slope (SS), *I*_ON_/*I*_OFF_ ratio, and sensitivity has been critically investigated to comment on the reliability and the sensitivity of the structure. Further, the junctionless nature of the proposed TFET sensor can result in reduced random dopant fluctuations (RDFs) and reduced thermal budget, and the sensor can be easily fabricated. A comparative analysis of the proposed biosensor with the state of the art sensors has shown a substantial improvement in the sensitivity of the FE-JL-TFET sensor, as it is 10^7^ which is many orders of magnitude higher than that of other previously reported biosensors.

The remainder of this paper is divided into three more sections. Section 2 discusses the proposed architecture and the models used in the simulation. Various results are thoroughly discussed in Section 3. Section 4 concludes the work with some important findings.

## 2 Device structure and simulation

**Fig. 1:**
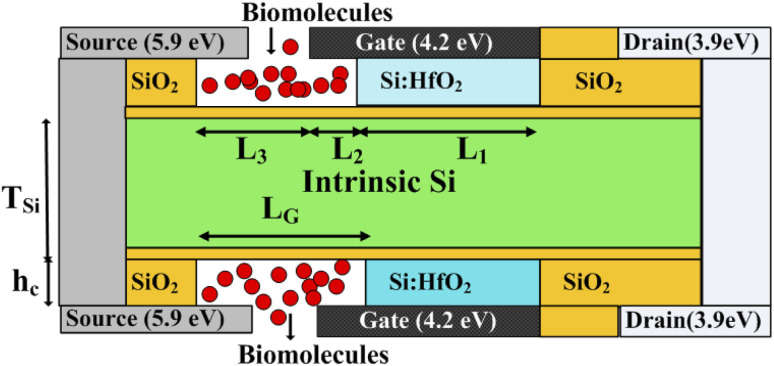
2-D schematic of the FE-JL-TFET based biosensor and pictorial depiction of biomolecule immobilization by the FE-JL-TFET based biosensor.

**Fig. 2:**
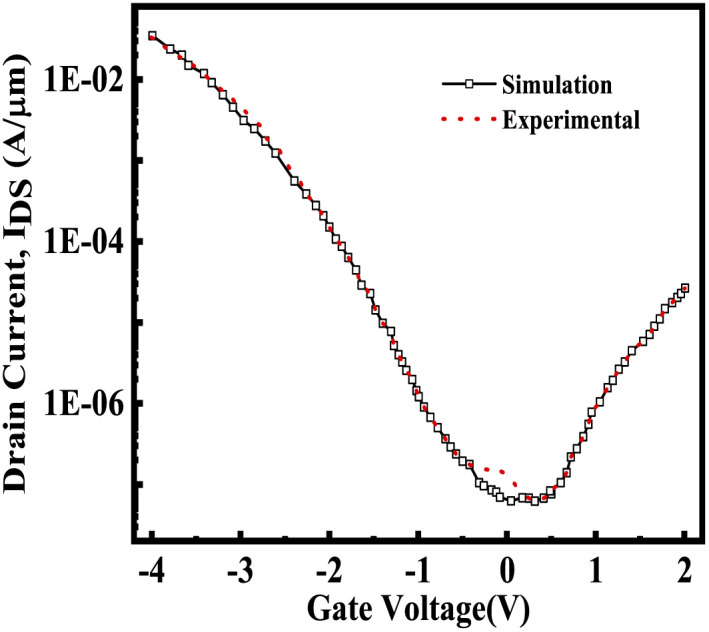
Calibration of the simulation model with a fabricated Fe-TFET based device.^18^

The schematic view of the proposed FE-JL-TFET biosensor and pictorial depiction of biomolecule immobilization by the FE-JL-TFET based biosensor are illustrated in Fig. 1. The nano-cavity carved in the source-gate dielectric acts as the binding site for the immobilization of the biomolecules by the device. The formation of the nano-cavity region with an inverted-T shape at the source-gate dielectric leads to an improved performance and suppression of ambipolarity and leakage current, improves the gate control and also improves the sensitivity. This region is used to detect the presence of bio-analytes with the effect of the dielectric modulation approach. The two cavities are carved in gate-source underlap regions by sacrificial etching. This method of sacrificial removal uses wet or dry etching depending on the commercially available HF chemicals. In order to create the hybrid air gap, a chemical agent in the liquid or gas phase sequentially etches the SiO_2_ layer.^46^ For the considered structure the source and drain (S/D) regions are realized on an ultra thin silicon film by deploying appropriate metal work-functions to act as electrodes. Moreover, the Si-doped HfO_2_ FE material is utilized as a gate stack dielectric to amplify the low gate voltage to enable the ultra steep switching, lower detection time and low power applications. The FE material (Si:HfO_2_) properties utilized for the device simulation are: coercive field (*F*_C_), remanent polarization (*P*_r_), saturation polarization (*P*_s_) and the dielectric constant of doped Si:HfO_2_ (FE) and values of 1 MV cm^−1^, 1 μC cm^−2^, 20 μC cm^−2^, and 31, respectively, have been chosen to introduce the negative capacitance effect.^31^ A hafnium electrode (work function = 3.9 eV) is introduced to create the N^+^ zone, which induces electrons at the drain side. Similar to this, platinum (work function = 5.93 eV) is employed in the source area by inducing holes with a concentration comparable to the P^+^ source doping of the reference device in the intrinsic silicon substrate.^23,30^Table 1 provides a list of the particular device dimension specifications used for the device emulation. There are two portions of the length of the gate metal dielectric underneath the gate metal: region 1 with length *L*_2_ comprises the gate underlap region which acts as a binding site for the biomolecules and region 2 comprises the gate dielectric with length *L*_1_. In this work, charged biomolecules with the concentration (*N*_f_) of *N*_f_ = 10^10^ cm^−2^ to *N*_f_ = 10^12^ cm^−2^ have been investigated. Various models have been incorporated during the TCAD ATLAS^33^ simulation using a calibrated exhaustive 2D simulation framework. The tunnel rate at the tunnel junction has been estimated in this circumstance using the non-local band-to-band tunneling model (non-local BTBT model). The CVT model is used to account for field and concentration dependent mobility. The Shockley–Read–Hall (SRH) model is included to account for carrier recombination. Studying the impact of high concentration on the bandgap is made possible by using the band gap narrowing (BGN) model. It is decided to incorporate the property changes of a strongly doped region using the Fermi–Dirac statistics model.^13–15^ The simulation study performed in this work is calibrated with the experimental data of a fabricated Fe-TFET reported by A. M. Ionescu *et al.*^21^Fig. 2 shows a perfect agreement between the results reported in ref. 21 and the simulated ones obtained using TCAD ATLAS.^33^

**Table:** Simulation parameters for the FE-JL-TFET based biosensor

Parameters	Value
Silicon thickness (*T*_Si_)	10 nm
Gate length (*L*_G_ = *L*_1_ + *L*_2_)	40 nm + 10 nm
Source length (*L*_S_)	100 nm
Drain length (*L*_D_)	100 nm
Background doping (*N*_in_)	1 × 10^16^ cm^−3^
Source work-function (*ϕ*_S_ (Pt))	5.93 eV
Gate-oxide thickness (*T*_ox_)	2.5 nm
Drain work-function (*ϕ*_D_ (Hf))	3.9 eV
Gate dielectric constant (*ε*_ox_)	HfO_2_ (31)
Cavity height (*h*_c_)	2.5 nm
Cavity length (*L*_c_ = *L*_2_ + *L*_3_)	10 nm + 30 nm

## 3 Results and discussions

In this section, the impact on the electrical characteristics of the device due to the presence of neutral biomolecules of different *K* values in the cavity region has been investigated. On the other hand, fixed charge is considered at the Si–SiO_2_ interface in case of the presence of charged biomolecules. Optimizing the cavity length (*L*_c_) and cavity height (*h*_c_) significantly optimizes the sensing capability of the proposed device. The cavity length (*L*_c_) and cavity height (*h*_c_) are selected according to the state of the art biosensors and they are in accordance with the biomolecule’s size.^34–39^

*K* = 1 reflects an empty cavity and the cavity filled with biomolecules (*K* > 1) shows a deviation in the device characteristics in comparison to the empty cavity, which has been utilized for the sensitivity analysis for different biomolecules.

### 3.1 Impact of cavity length (*L*_c_) variation

**Fig. 3:**
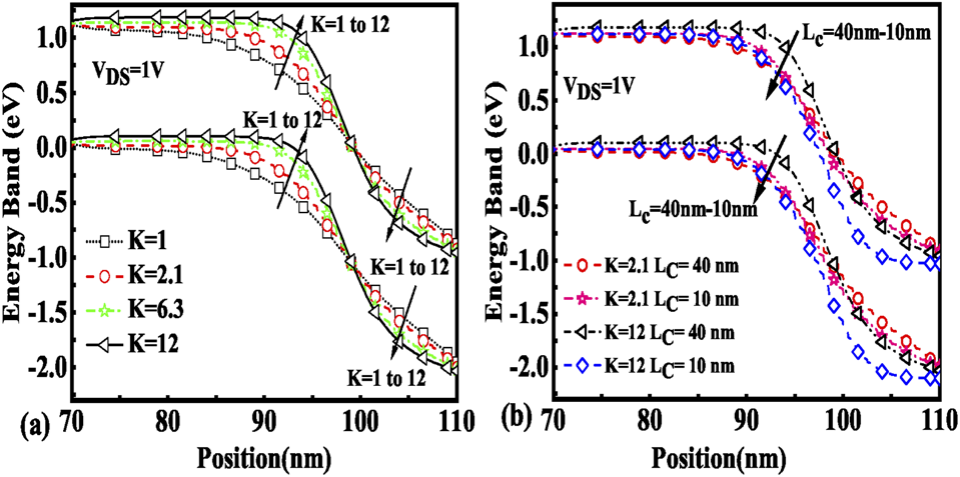
Energy band diagram of the FE-JL-TFET based biosensor (a) for neutral biomolecules and (b) with cavity length *L*_c_ variations for neutral biomolecules.

**Fig. 4:**
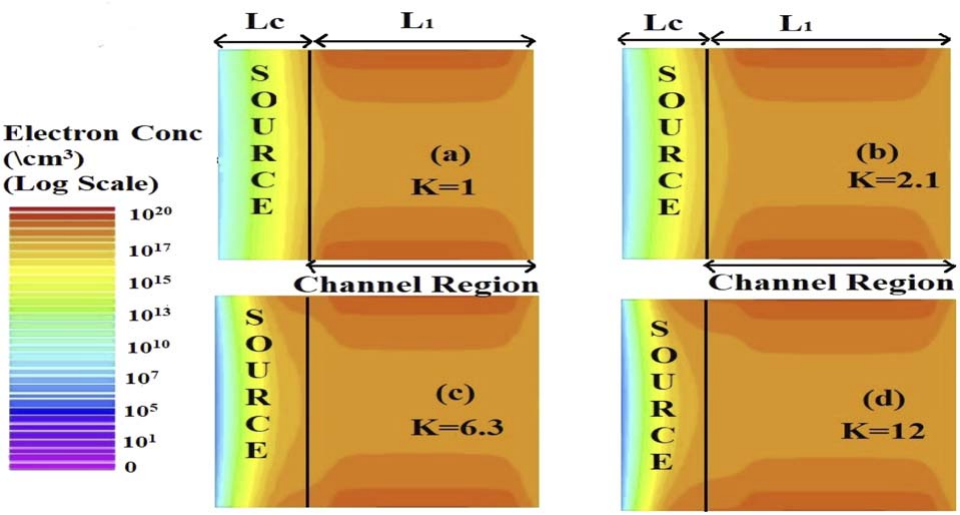
2-D electron density variation in the Si channel region and cavity region in the ON-state (*V*_GS_ = 1 V, *V*_DS_ = 1 V) for different dielectric constants (a) *K* = 1, (b) *K* = 2.1, (c) *K* = 6.3, and (d) *K* = 12.

**Fig. 5:**
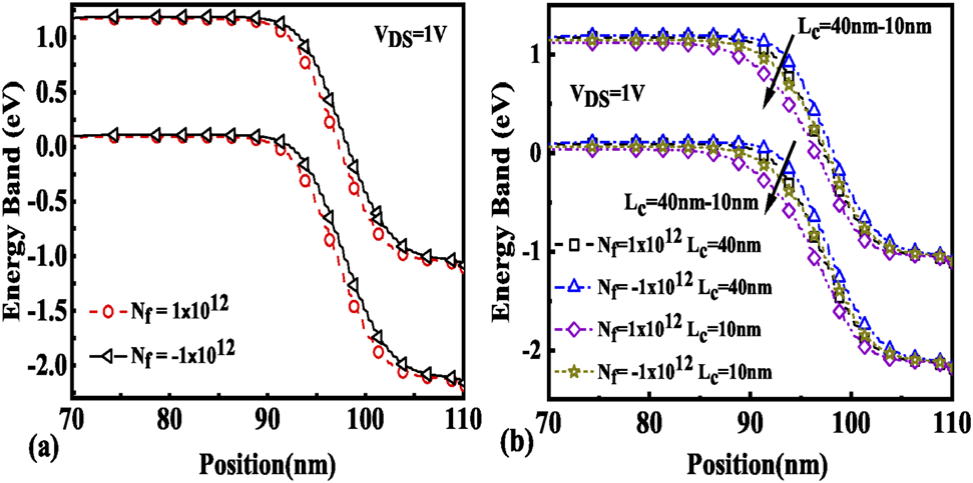
Energy band diagram of the FE-JL-TFET based biosensor (a) for charged biomolecules and (b) with cavity length variations for charged biomolecules.

The effect of immobilization of neutral biomolecules in the cavity region on the energy band diagram is shown in Fig. 3(a). It is observed that creating a cavity in the structure results in a larger barrier width between the channel conduction band and the source valence band and hence reduces the carrier tunneling probability. It has also been observed that an increase in *K* leads to more energy band bending which further reduces the barrier width.^26^Fig. 3(b) shows the impact of cavity length variation on the energy band for dielectric values *K* = 2.1 and *K* = 12. Increasing the dielectric constant from unity to higher values with the immobilization of the biomolecules in the cavity region leads to enhanced electron density as shown in Fig. 4. However, the impact of charged biomolecules on the energy band diagram of the FE-JL-TFET with the nanogap cavity is depicted in Fig. 5(a). It can be noted that the immobilization of positively (negatively) charged biomolecules at the binding sites results in a decrease (increase) in the barrier width between the channel conduction band (CB) and the valence band (VB) of the source. It is also observed from Fig. 5(b) that varying the length of the cavity from 40 nm to 10 nm increases the barrier width between the CB and VB along the channel length from the source to the drain region.

**Fig. 6:**
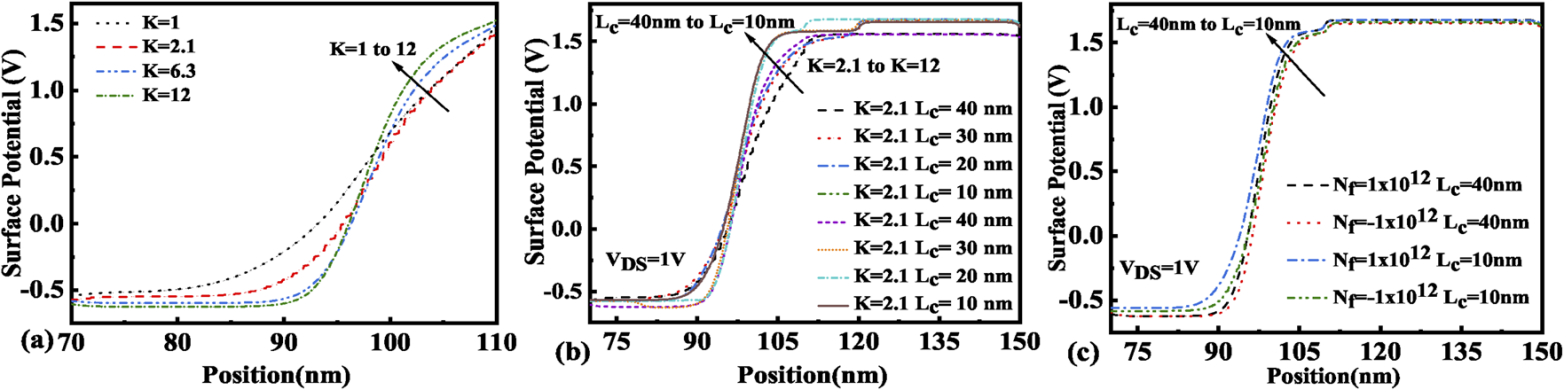
Surface potential of the FE-JL-TFET based biosensor (a) for neutral biomolecules, (b) with cavity length variations for neutral biomolecules, and (c) with cavity length variations for charged biomolecules.

The immobilization of neutral biomolecules with *K* value varying from *K* = 2.1 to 12 in the nano-cavity region results in an enhanced gate capacitance and therefore results in an improvement in the surface potential for the FE-JL-TFET as shown in Fig. 6(a). The enhancement in the effective gate capacitance due to the increase in *K* value also reduces the barrier at the source–channel junction, which further increases the surface potential in the cavity region. With the formation of the nano-cavity region in the structure, the barrier between the source and the channel is enhanced. It is found that the surface potential of the device increases with a reduction in the cavity length (*L*_c_). As the length of the cavity is reduced from 40 nm to 10 nm, the change in the surface potential is depicted in Fig. 6(b). The impact of immobilized charged biomolecules in the cavity region on the surface potential is depicted in Fig. 6(c). An increment in the surface potential is achieved for positively charged biomolecules (*N*_f_ = 1 × 10^12^ cm^−2^) trapped in the cavity region with *K* = 12.^30^

**Fig. 7:**
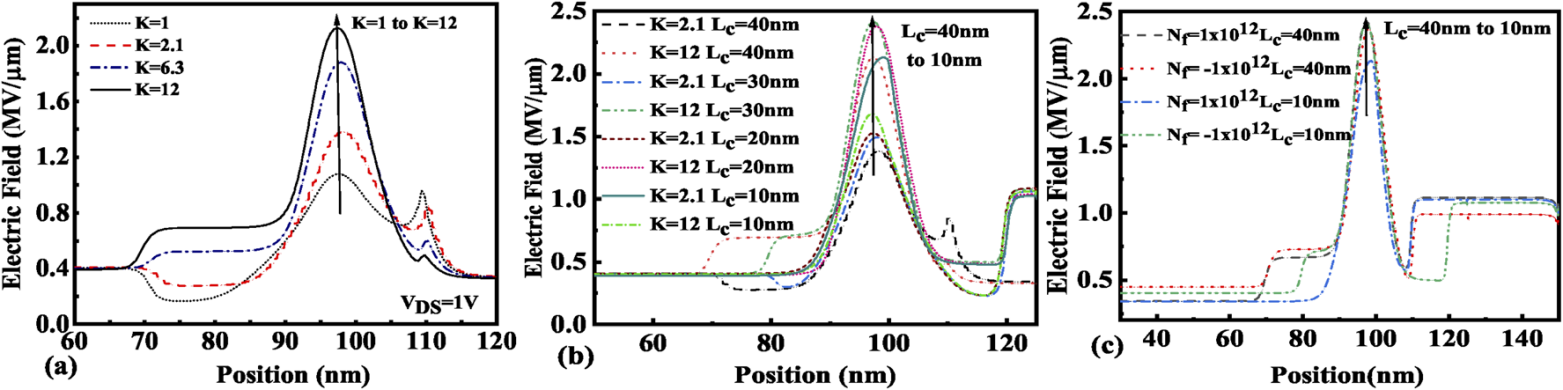
Electric field of the FE-JL-TFET based biosensor (a) for neutral biomolecules, (b) with cavity length variations for neutral biomolecules, and (c) with cavity length variations for charged biomolecules.

**Fig. 8:**
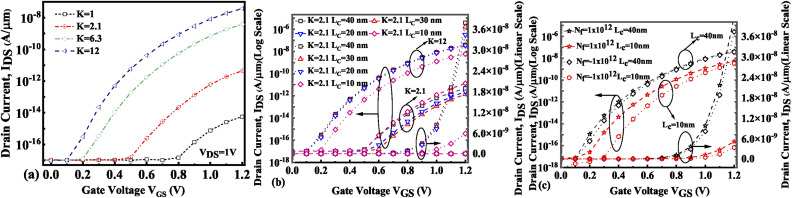
Transfer characteristics of the FE-JL-TFET based biosensor (a) for neutral biomolecules, (b) with cavity length *L*_c_ variations for neutral biomolecules, and (c) with cavity length *L*_c_ variations for charged biomolecules.

The presence of negatively charged biomolecules (*N*_f_ = −1 × 10^12^ cm^−2^) leads to an increment in the flat band voltage (*V*_fb_) achieved during the presence of negatively charged biomolecules in the cavity region, which results in reduced effective gate bias. This further reduces the surface potential in the nano-cavity region as shown in Fig. 6(c). The negatively charged biomolecule immobilization leads to an increase in the tunneling barrier width as well as reduced surface potential. However, positively charged biomolecules follow the opposite behavior. Band bending of the structure is achieved at a higher value of *K* as depicted in Fig. 3(a) and an enhanced electric field can be observed at a higher value of *K* as shown in Fig. 7(a). Further, the impact of variation of *L*_c_ on the electric field for neutral as well as charged biomolecules is depicted in Fig. 7(b) and (c), respectively. It can be noted that at lower dielectric constant, *i.e.* for *K* = 2.1, EF reduces with an increase in the cavity length as the gate capacitance starts diminishing at higher cavity length. On the other hand, for higher dielectric constant, *i.e.* for *K* = 12, the reverse phenomenon takes place. The transfer characteristic variations for different neutral biomolecules are depicted in Fig. 8(a). It has been noted that the tunneling barrier width modulates as the hole carrier density rises with the dielectric constant in the cavity area.

**Fig. 9:**
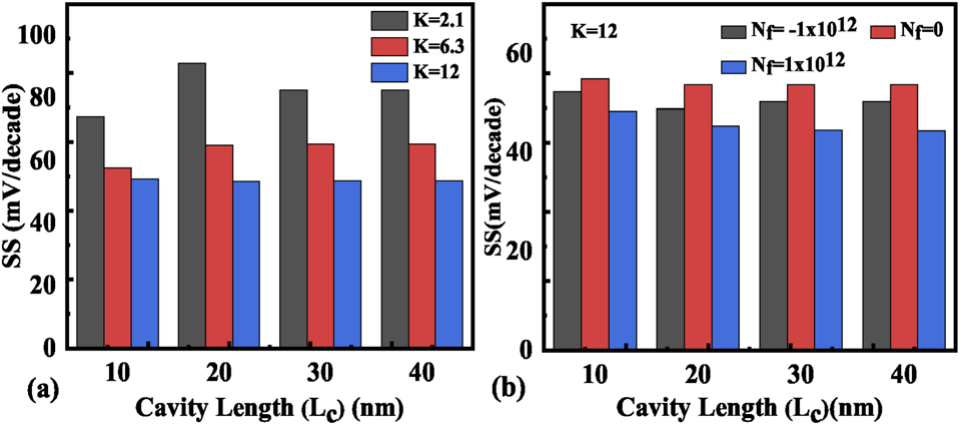
Subthreshold slope of the FE-JL-TFET based biosensor (a) with cavity length *L*_c_ variations for neutral biomolecules, (b) with cavity length *L*_c_ variations for charged biomolecules.

Hence, an increase in Wentzel–Kramers–Brillouin tunneling probability (*T*_WKB_) leads to a higher ON current. Fig. 8(b) and (c) depict the impact of cavity length variation on the transfer characteristics with neutral biomolecules and charged biomolecules respectively. It can be seen that an increase in the cavity length results in enhanced drive current owing to the carrier modulation (due to increased electric field) under the cavity region as depicted in Fig. 7(b) with cavity length variation from *L*_c_ = 10 nm to 40 nm for *K* = 12. For *K* = 2.1 the reverse impact of cavity length variation on drain current has been observed. The impact of charged biomolecule concentrations captured in the cavity region on the transfer characteristics with a variation in cavity length is depicted in Fig. 8(c). It is observed that a decrease in cavity length reduces the drive current for both positively and negatively charged biomolecules. The SS variation with the cavity length for various dielectric constants such as *K* = 2.1, 6.3 and 12 is depicted in Fig. 9(a). It is found that with an increase in the dielectric constant the SS value follows a decreasing trend.^31^ It is observed from Fig. 9(a) that the value of SS attains a minimum at *L*_c_ = 10 nm. The increased gate control over the channel causes a rise in biomolecule confluence, which lowers SS with rising *K* value. Fig. 9(b) presents the variation in SS with an increase in the cavity length for charged biomolecules immobilized in the cavity region. From the figure it is evident that on varying the cavity length from 10 nm to 40 nm, the SS value reduced on enhancing the biomolecules’ relative permittivity inside the cavity. A large cavity length *L*_c_ shows a large variation in SS. Table 2 shows the *I*_ON_, *I*_OFF_, and *I*_ON_/*I*_OFF_ ratio of neutral biomolecules for different cavity lengths. It is evident that with an increase in *K* value, the *I*_ON_/*I*_OFF_ ratio increases as *I*_ON_ increases but *I*_OFF_ remains constant. It follows the same trend as reported in ref. 36, 40 and 41. Here, it is worth mentioning that *I*_ON_ increases for *K* = 1 to 12 but not linearly. Actually, we do not even expect analytically exact linear variations as the reported device is a ferroelectric TFET. The result we are getting is very much in accordance with the existing state-of-the-art as included in ref. 34, 41 and 42. Tables 3 and 4 present the *I*_ON_, *I*_OFF_, and *I*_ON_/*I*_OFF_ ratio for positively charged and negatively charged biomolecules of different dielectric constant values at different cavity lengths. It can be observed that at higher cavity length and at higher biomolecule charge the *I*_ON_/*I*_OFF_ ratio is higher and it is higher for negatively charged biomolecules than positively charged biomolecules.

Variation in *I*_ON_, *I*_OFF_, and *I*_ON_/*I*_OFF_ ratio for different neutral biomolecules when the length of the cavity varies

**Table:** 

Dielectric constant (*K*)	Cavity length (*L*_c_ = 10 nm)			Cavity length (*L*_c_ = 20 nm)		
	*I*_ON_	*I*_OFF_	*I*_ON_/*I*_OFF_	*I*_ON_	*I*_OFF_	*I*_ON_/*I*_OFF_
*K* = 1	1.44 × 10^−13^	9.97 × 10^−18^	1.45 × 10^4^	3.23 × 10^−15^	9.97 × 10^−18^	3.24 × 10^2^
*K* = 2.1	1.29 × 10^−11^	1 × 10^−17^	1.28 × 10^6^	2.37 × 10^−12^	9.97 × 10^−18^	2.37 × 10^5^
*K* = 6.3	1.2 × 10^−9^	9.97 × 10^−18^	1.2 × 10^8^	3.61 × 10^−9^	9.97 × 10^−18^	3.62 × 10^8^
*K* = 12	5.78 × 10^−9^	9.97 × 10^−18^	5.8 × 10^8^	3.53 × 10^−8^	9.97 × 10^−18^	3.54 × 10^9^

**Table:** 

	Cavity length (*L*_c_ = 30 nm)			Cavity length (*L*_c_ = 40 nm)		
	*I*_ON_	*I*_OFF_	*I*_ON_/*I*_OFF_	*I*_ON_	*I*_OFF_	*I*_ON_/*I*_OFF_
*K* = 1	7.16 × 10^−15^	9.97 × 10^−18^	7.18 × 10^2^	5.75 × 10^−15^	9.97 × 10^−18^	5.76 × 10^2^
*K* = 2.1	4.5 × 10^−12^	9.97 × 10^−18^	4.51 × 10^5^	4.47 × 10^−12^	1.05 × 10^−17^	4.24 × 10^5^
*K* = 6.3	3.89 × 10^−9^	9.97 × 10^−18^	3.9 × 10^8^	3.89 × 10^−9^	9.97 × 10^−18^	3.9 × 10^8^
*K* = 12	3.77 × 10^−8^	9.97 × 10^−18^	3.77 × 10^9^	3.76 × 10^−8^	9.97 × 10^−18^	3.77 × 10^9^

**Table:** Variation in *I*_ON_, *I*_OFF_, and *I*_ON_/*I*_OFF_ ratio for different positively charged biomolecules when the length of the cavity varies

Dielectric constant (*K*)	*N*_f_	Cavity length (*L*_c_ = 40 nm)			Cavity length (*L*_c_ = 10 nm)		
		*I*_ON_	*I*_OFF_	*I*_ON_/*I*_OFF_	*I*_ON_	*I*_OFF_	*I*_ON_/*I*_OFF_
*K* = 2.1	1 × 10^10^	4.47 × 10^−12^	7.02 × 10^−18^	6.37 × 10^5^	4.23 × 10^−11^	7.07 × 10^−18^	5.98 × 10^6^
	1 × 10^11^	4.47 × 10^−12^	8.72 × 10^−18^	5.13 × 10^5^	4.39 × 10^−11^	8.87 × 10^−18^	4.95 × 10^6^
	1 × 10^12^	4.21 × 10^−12^	6.5 × 10^−18^	6.48 × 10^5^	6.13 × 10^−11^	6.55 × 10^−18^	9.36 × 10^6^
*K* = 6.3	1 × 10^10^	3.88 × 10^−9^	7.25 × 10^−18^	5.35 × 10^8^	1.35 × 10^−9^	7.2 × 10^−18^	1.87 × 10^8^
	1 × 10^11^	3.93 × 10^−9^	9.86 × 10^−18^	3.98 × 10^8^	1.35 × 10^−9^	8.77 × 10^−18^	1.54 × 10^8^
	1 × 10^12^	4.42 × 10^−9^	6.5 × 10^−18^	6.8 × 10^8^	1.32 × 10^−9^	6.55 × 10^−18^	2.01 × 10^8^
*K* = 12	1 × 10^10^	3.76 × 10^−8^	7.26 × 10^−18^	5.18 × 10^9^	5.94 × 10^−9^	7.25 × 10^−18^	8.18 × 10^8^
	1 × 10^11^	3.79 × 10^−8^	8.98 × 10^−18^	4.22 × 10^9^	5.86 × 10^−9^	9.92 × 10^−18^	5.98 × 10^8^
	1 × 10^12^	4.14 × 10^−8^	7.07 × 10^−18^	5.85 × 10^9^	5.05 × 10^−9^	7.02 × 10^−18^	7.19 × 10^8^

**Table:** Variation in *I*_ON_, *I*_OFF_, and *I*_ON_/*I*_OFF_ ratio for different negatively charged biomolecules when the length of the cavity varies

Dielectric constant (*K*)	*N*_f_	Cavity length (*L*_c_ = 40 nm)			Cavity length (*L*_c_ = 10 nm)		
		*I*_ON_	*I*_OFF_	*I*_ON_/*I*_OFF_	*I*_ON_	*I*_OFF_	*I*_ON_/*I*_OFF_
*K* = 2.1	−1 × 10^10^	4.46 × 10^−12^	8.01 × 10^−18^	5.57 × 10^5^	4.2 × 10^−11^	1.33 × 10^−17^	3.14 × 10^6^
	−1 × 10^11^	4.45 × 10^−12^	5.53 × 10^−18^	8.04 × 10^5^	4.04 × 10^−11^	6.05 × 10^−18^	6.68 × 10^6^
	−1 × 10^12^	4.08 × 10^−12^	2.22 × 10^−18^	1.83 × 10^6^	2.73 × 10^−11^	1.54 × 10^−18^	1.77 × 10^7^
*K* = 6.3	−1 × 10^10^	3.87 × 10^−9^	7.41 × 10^−18^	5.35 × 10^8^	1.35 × 10^−9^	9.74 × 10^−18^	1.38 × 10^8^
	−1 × 10^11^	3.82 × 10^−9^	6.6 × 10^−18^	5.78 × 10^8^	1.35 × 10^−9^	5.53 × 10^−18^	2.43 × 10^8^
	−1 × 10^12^	3.27 × 10^−9^	2.22 × 10^−18^	1.47 × 10^9^	1.3 × 10^−9^	1.93 × 10^−18^	6.74 × 10^8^
*K* = 12	−1 × 10^10^	3.75 × 10^−8^	6.99 × 10^−18^	5.36 × 10^9^	5.95 × 10^−9^	1.01 × 10^−17^	5.89 × 10^8^
	−1 × 10^11^	3.71 × 10^−8^	5.53 × 10^−18^	6.71 × 10^9^	6.02 × 10^−9^	6 × 10^−18^	1 × 10^9^
	−1 × 10^12^	3.33 × 10^−8^	1.93 × 10^−18^	1.72 × 10^10^	6.61 × 10^−9^	2.21 × 10^−18^	2.9 × 10^9^

### 3.2 Impact of cavity height (*h*_c_) variation

**Fig. 10:**
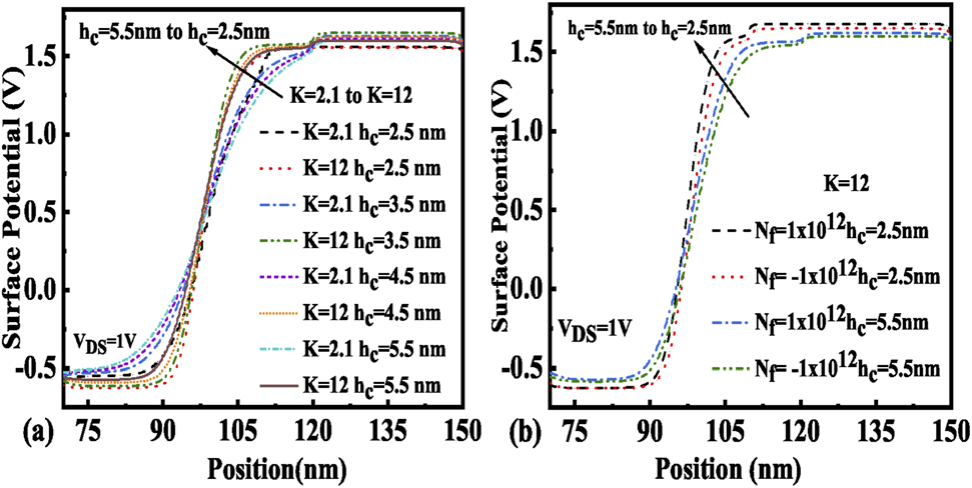
Surface potential of the FE-JL-TFET based biosensor (a) with cavity height *h*_c_ variations for neutral biomolecules, (b) with cavity height *h*_c_ variations for charged biomolecules.

In the design of biosensor devices, it is also crucial to optimize the cavity height. Thus, the effect of variation of *h*_c_ on various electrical performance parameters has been studied in this subsection. The impact of *h*_c_ variation on surface potential for various neutral and charged biomolecules is illustrated in Fig. 10(a) and (b), respectively. It is found that with an increase in *h*_c_, the surface potential of the device under the cavity region falls. It has also been noticed that the surface potential variation throughout the channel length reduces from the source end to the drain end with *h*_c_ increasing from 2.5 nm to 5.5 nm. Fig. 10(a) shows that the maximum value of surface potential was achieved at *h*_c_ = 2.5. Fig. 10(b) depicts the surface potential variation due to charged biomolecules trapped in the cavity region. It shows that the surface potential increases sharply due to positively charged biomolecules trapped in the cavity region and reduces due to the presence of negatively charged biomolecules in the cavity region. This indicates that immobilization of positively charged biomolecules results in an increase in the band bending, which further reduces the barrier width and hence results in an increase in the surface potential.

**Fig. 11:**
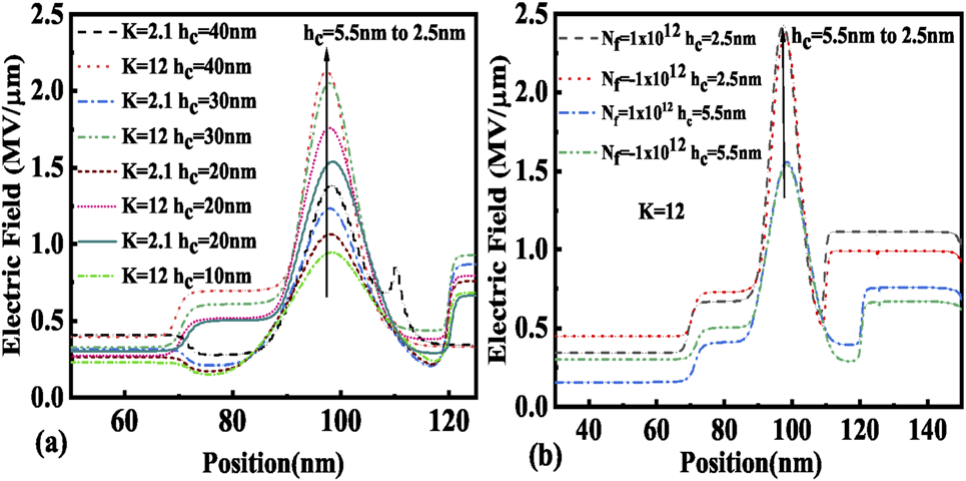
Electric field of the FE-JL-TFET based biosensor (a) with cavity height *h*_c_ variations for neutral biomolecules and (b) with cavity height *h*_c_ variations for charged biomolecules.

The variation in electric field with cavity height is depicted in Fig. 11(a) and (b) for neutral and charged biomolecules respectively. It is observed that an increase in *h*_c_ from 2.5 nm to 5.5 nm leads to lowering of the gate control over the channel region, hence there is a degradation in EF observed for both neutral and charged biomolecules. It has also been noticed that positively charged biomolecules have a larger EF than negatively charged biomolecules.

**Fig. 12:**
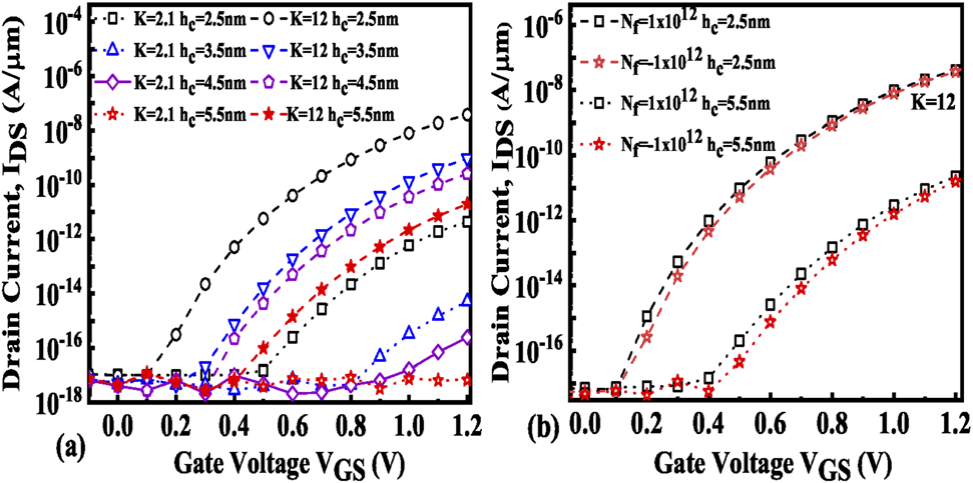
Transfer characteristics of the FE-JL-TFET based biosensor (a) with cavity height *h*_c_ variations for neutral biomolecules and (b) with cavity height *h*_c_ variations for charged biomolecules.

**Fig. 13:**
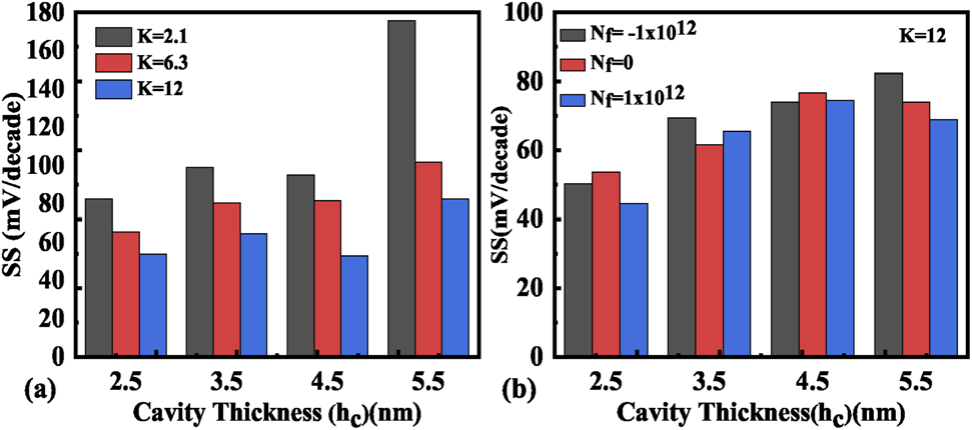
Subthreshold slope of the FE-JL-TFET based biosensor (a) with cavity height *h*_c_ variations for neutral biomolecules and (b) with cavity height variations for charged biomolecules.

The impact of cavity height variation on the transfer characteristics is depicted in Fig. 12(a). It can be seen that an increase in the cavity height reduces the drive current as the EF is degraded as the cavity height increases. The impact of charged biomolecule concentrations immobilized in the cavity region on the transfer characteristic with a variation in cavity height is depicted in Fig. 12(b). It is observed that an increase in the cavity height from 2.5 nm to 5.5 nm leads to a reduction in the drive current of almost three decades for both positively and negatively charged biomolecules. The SS variation with the different cavity heights for various dielectric constants such as *K* = 2.1, 6.3 and 12 is depicted in Fig. 13(a). It is observed that the value of SS attains a minimum value for the cavity height of 2.5 nm. The gate control over the channel region is reduced with an increase in cavity height which leads to an increase in the SS value. Fig. 13(b) presents the variation in SS with an increase in the cavity height for charged biomolecules immobilized in the cavity region. It can be noted that on changing the cavity height from 2.5 nm to 5.5 nm there is an increment in the SS value. Table 5 shows the *I*_ON_, *I*_OFF_, and *I*_ON_/*I*_OFF_ ratio of neutral biomolecules for different cavity heights. Table 6 presents the *I*_ON_, *I*_OFF_, and *I*_ON_/*I*_OFF_ ratio for charged biomolecules of different dielectric constants at different cavity heights. It is evident that at lower cavity height and higher *K* value (charge as well) the *I*_ON_/*I*_OFF_ ratio is higher and it is even higher for negatively charged biomolecules compared to positively charged biomolecules.

Variation in *I*_ON_, *I*_OFF_, and *I*_ON_/*I*_OFF_ ratio for different neutral biomolecules when the cavity height varies

**Table:** 

Dielectric constant (*K*)	Cavity height (*h*_c_ = 5.5 nm)			Cavity height (*h*_c_ = 4.5 nm)		
	*I*_ON_	*I*_OFF_	*I*_ON_/*I*_OFF_	*I*_ON_	*I*_OFF_	*I*_ON_/*I*_OFF_
*K* = 2.1	6.74 × 10^−18^	4.38 × 10^−18^	1.58	2.44 × 10^−16^	4.12 × 10^−18^	59.2
*K* = 6.3	4.48 × 10^−13^	4.38 × 10^−18^	1.02 × 10^5^	8.71 × 10^−12^	3.76 × 10^−18^	2.32 × 10^6^
*K* = 12	1.98 × 10^−11^	4.43 × 10^−18^	4.47 × 10^6^	2.62 × 10^−10^	4.12 × 10^−18^	6.35 × 10^7^

**Table:** 

	Cavity height (*h*_c_ = 3.5 nm)			Cavity height (*h*_c_ = 2.5 nm)		
	*I*_ON_	*I*_OFF_	*I*_ON_/*I*_OFF_	*I*_ON_	*I*_OFF_	*I*_ON_/*I*_OFF_
*K* = 2.1	5.15 × 10^−15^	4.85 × 10^−18^	1.06 × 10^3^	4.47 × 10^−12^	1.05 × 10^−17^	4.24 × 10^5^
*K* = 6.3	5.14 × 10^−11^	5.09 × 10^−18^	1 × 10^7^	3.89 × 10^−9^	9.97 × 10^−18^	3.9 × 10^8^
*K* = 12	9.58 × 10^−10^	5.09 × 10^−18^	1.88 × 10^8^	3.76 × 10^−8^	9.97 × 10^−18^	3.77 × 10^9^

**Table:** Variation in *I*_ON_, *I*_OFF_, and *I*_ON_/*I*_OFF_ ratio for different charged biomolecules when the cavity height varies

Dielectric constant (*K*)	*N*_f_	Cavity height (*h*_c_ = 5.5 nm)			Cavity height (*h*_c_ = 2.5 nm)		
		*I*_ON_	*I*_OFF_	*I*_ON_/*I*_OFF_	*I*_ON_	*I*_OFF_	*I*_ON_/*I*_OFF_
*K* = 2.1	0	9.8 × 10^−18^	6.03 × 10^−18^	1.63	4.46 × 10^−12^	7.04 × 10^−18^	6.33 × 10^5^
	1 × 10^12^	7.02 × 10^−18^	4.38 × 10^−18^	1.6	4.21 × 10^−12^	6.5 × 10^−18^	6.48 × 10^5^
	−1 × 10^12^	7.75 × 10^−18^	3.73 × 10^−18^	2.07	4.08 × 10^−12^	2.22 × 10^−18^	1.83 × 10^6^
*K* = 12	0	1.97 × 10^−11^	6.81 × 10^−18^	2.9 × 10^6^	3.7 × 10^−8^	7.12 × 10^−17^	5.25 × 10^9^
	1 × 10^12^	2.3 × 10^−11^	4.9 × 10^−18^	4.7 × 10^6^	4.14 × 10^−8^	7.07 × 10^−18^	5.85 × 10^9^
	−1 × 10^12^	1.61 × 10^−11^	4.67 × 10^−18^	3.46 × 10^6^	3.33 × 10^−8^	1.93 × 10^−18^	1.72 × 10^10^

**Fig. 14:**
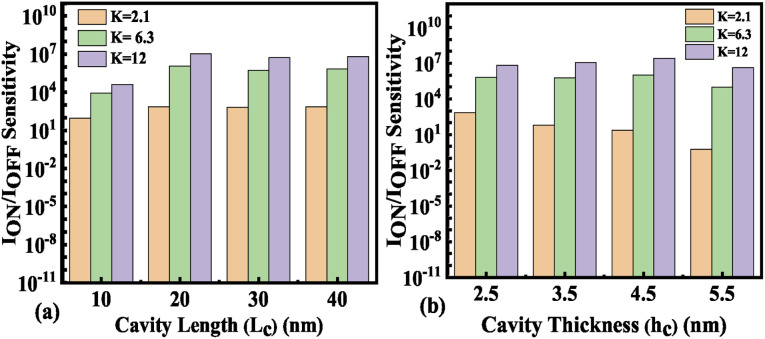
*I*_ON_/*I*_OFF_ sensitivity of the FE-JL-TFET based biosensor (a) with cavity length *L*_c_ variations for neutral biomolecules and (b) with cavity height variations for neutral biomolecules.

Fig. 14(b) shows the *I*_ON_/*I*_OFF_ sensitivity for neutral biomolecules with the variation of cavity height (*h*_c_) from 2. 5 nm to 5.5 nm. The maximum value of the *I*_ON_/*I*_OFF_ sensitivity is achieved as 3.77 × 10^9^ at *K* = 12 for *L*_c_ = 40 nm and *h*_c_ = 2.5 nm. For positively charged biomolecules the maximum value of the *I*_ON_/*I*_OFF_ sensitivity is achieved as 5.85 × 10^9^ at *N*_f_ = 1 × 10^12^ for *L*_c_ = 40 nm and *h*_c_ = 2.5 nm. For negatively charged biomolecules the maximum value of the *I*_ON_/*I*_OFF_ sensitivity is achieved as 1.72 × 10^10^ for *N*_f_ = −1 × 10^12^ at *L*_c_ = 40 nm and *h*_c_ = 2.5 nm. However, the maximum *I*_ON_/*I*_OFF_ sensitivity achieved is reported to be of the order of 10^9^ and 10^8^, respectively as reported in ref. 32 and 33. Thus the obtained *I*_ON_/*I*_OFF_ sensitivity is 10× and 100× higher than the earlier reported values. The detailed bench marking of the FET based biosensors is presented in Table 7.

**Table:** Bench marking for the FE-JL-TFET based biosensor

S. no.	FET based biosensors	Sensitivity (*I*_ON_/*I*_OFF_)	Sensitivity (*V*_th_)
1	Short-gate dielectric modulated electrically doped TFET^43^	10^5^	—
2	Vertical dielectrically modulated TFET^42^	10^1^	—
3	Dielectrically modulated junctionless TFET^12^	10^2^	—
4	Charge-plasma gate underlap doping-less TFET^14^	10^4^	—
5	Triple gate doping-less vertical TFET^34^	10^4^	—
6	Full-gate tunnel FET^10^	10^5^	—
7	Double gate junctionless FET^44^	—	400
8	Junctionless gate stack surrounding gate MOSFET^45^	—	190
9	Short-gate TFET^10^	10^6^	—
10	Graphene FET^47^	—	250
11	Present work	10^7^	720

**Fig. 15:**
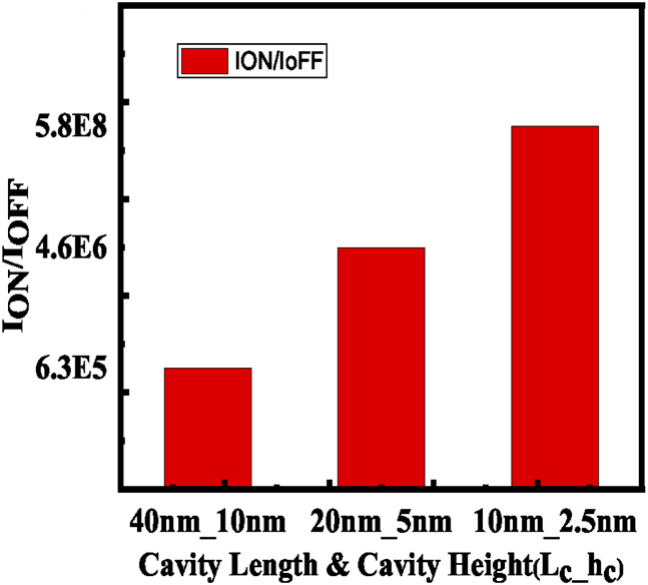
*I*_ON_/*I*_OFF_ current ratio with maintaining the aspect ratio (cavity length to cavity height) as a constant.

Devices with different cavity size were examined while the aspect ratio (cavity height to length) was kept constant. It has been found that keeping the aspect ratio constant and increasing both the cavity height and length results in a diminished value of *I*_ON_/*I*_OFF_ ratio. This is because the increase in cavity height results in reduced gate controllability as shown in Fig. 15.

## 4 Conclusion

The present work reports an ultra sensitive, label-free, and dielectric/charge modulated Si:HfO_2_ ferroelectric junctionless TFET (FE-JL-TFET) for biosensing applications. The negative capacitance technique due to the incorporated Si-doped HfO_2_ ferroelectric material and the dual inverted-T cavity carved in the source-gate underlap regions are the key enablers of the biosensing phenomenon. The inverted-T shape cavity at the source-gate dielectric leads to an improved performance and suppression of ambipolarity and leakage current, improves the gate control and also improves the sensitivity. By observing the impact of immobilized biomolecules in the cavity region on the electrical performance parameters of the biosensor, biomolecules are sensed. It is found that the reported biosensor exhibits lower leakage current and improved ON current, and the obtained sensitivities are much better for neutral as well as charged biomolecules. The maximum value of *I*_ON_/*I*_OFF_ sensitivity for neutral biomolecules is reported as 3.77 × 10^9^, that for positively charged biomolecules is 5.85 × 10^9^, and that for negatively charged biomolecules is 1.72 × 10^10^. Our research shows that cavity length and cavity height are the two most important parameters for improving biosensor sensitivity. Thus, the FE-JL-TFET demonstrates superior performance by achieving better sensitivity for both neutral and charged biomolecules by appropriate selection of the cavity length and cavity thickness near the source–channel junction. Our biosensor realizes several benefits such as label-free detection, higher *I*_ON_/*I*_OFF_ ratio, ultra steep SS, ultra low power applications, *etc.* Furthermore, the realization of a junctionless TFET results in less complex process technology, lower thermal budget, and reduced random dopant fluctuations (RDFs).

## Conflicts of interest

There are no conflicts to declare.

## Supplementary Material
